# Disability and Relapse Risk in Late-Onset Myelin Oligodendrocyte Glycoprotein Antibody–Associated Disease

**DOI:** 10.1001/jamanetworkopen.2025.59471

**Published:** 2026-02-13

**Authors:** Hyunjin Ju, Ki Hoon Kim, Sook Young Woo, Yeon Hak Chung, Ho Jin Kim, Hyunjin Kim, Eun-Jae Lee, Young-Min Lim, Woohee Ju, Sung-Min Kim, Young Nam Kwon, Seung Woo Kim, Ha Young Shin, In Soo Joo, Sohyeon Kim, Hung Youl Seok, Jeong Bin Bong, Byeol-A. Yoon, Jong Kuk Kim, You-Ri Kang, Tai-Seung Nam, Sooyoung Kim, Eunhee Sohn, Woojun Kim, Jin Myoung Seok, Hyung-Soo Lee, Sun-Young Oh, Suk-Won Ahn, Sukyoon Lee, Tae-Kyeong Lee, Hye Lim Lee, Nam-Hee Kim, Jeeyoung Oh, Jee-Eun Kim, Soonwook Kwon, Seong-il Oh, Min Su Park, Jong Seok Bae, Wookyung Kim, Jin-Woo Park, Byung-Jo Kim, Jiwon Yang, Su-Hyun Kim, Ju-Hong Min

**Affiliations:** 1 Department of Neurology, Samsung Medical Center, Sungkyunkwan University School of Medicine, Seoul, Republic of Korea; 2 Department of Neurology, Soonchunhyang University Seoul Hospital, Soonchunhyang University College of Medicine, Seoul, Republic of Korea; 3 Department of Neurology, Research Institute and Hospital of National Cancer Center, Goyang, Republic of Korea; 4 Department of Neurology, Severance Hospital, Yonsei University College of Medicine, Seoul, Republic of Korea; 5 Biomedical Statistics Center, Research Institute for Future Medicine, Samsung Medical Center, Republic of Korea; 6 Department of Neurology, Korea University Guro Hospital, Korea University College of Medicine, Seoul, Republic of Korea; 7 Department of Neurology, Asan Medical Center, University of Ulsan College of Medicine, Seoul, Republic of Korea; 8 Department of Neurology, Seoul National University Hospital, Seoul National University College of Medicine, Seoul, Republic of Korea; 9 Department of Neurology, Chung-Ang University Hospital, Seoul, Republic of Korea; 10 Department of Neurology, Ajou University School of Medicine, Suwon, Republic of Korea; 11 Department of Neurology, Dongsan Hospital, Keimyung University School of Medicine, Daegu, Republic of Korea; 12 Department of Neurology, School of Medicine, Kyungpook National University, Daegu, Republic of Korea; 13 Department of Neurology, Chosun University College of Medicine, Gwangju, Republic of Korea; 14 Department of Neurology, Dong-A University College of Medicine, Busan, Republic of Korea; 15 Department of Neurology, Chonnam National University Hospital, Chonnam National University Medical School, Gwangju, Republic of Korea; 16 Department of Neurology, Chungnam National University College of Medicine, Chungnam National University Hospital, Daejeon, Republic of Korea; 17 Department of Neurology, Seoul St Mary’s Hospital, The Catholic University of Korea, Seoul, Republic of Korea; 18 Department of Neurology, Ewha Womans University Seoul Hospital, Ewha Womans University College of Medicine, Seoul, Republic of Korea; 19 Department of Neurology, National Medical Center, Seoul, Republic of Korea; 20 Department of Neurology, Jeonbuk National University School of Medicine, Jeonju, Republic of Korea; 21 Department of Neurology, Busan Paik Hospital, Inje University College of Medicine, Busan, Republic of Korea; 22 Department of Neurology, Soonchunhyang University Bucheon Hospital, Bucheon, Republic of Korea; 23 Department of Neurology, Ilsan Hospital, Dongguk University College of Medicine, Goyang, Republic of Korea; 24 Department of Neurology, Konkuk University Medical Center, Seoul, Republic of Korea; 25 Department of Neurology, Inha University Hospital, Inha University College of Medicine, Incheon, Republic of Korea; 26 Department of Neurology, Kyung Hee University Hospital, Kyung Hee University School of Medicine, Seoul, Republic of Korea; 27 ParkMinSu Neurology Clinic, Daegu, Republic of Korea; 28 Department of Neurology, Kangdong Sacred Heart Hospital, Hallym University College of Medicine, Seoul, Republic of Korea; 29 Department of Neurology, Korea University College of Medicine, Seoul, Republic of Korea; 30 Department of Neurology, Gil Medical Center, Gachon University College of Medicine, Incheon, Republic of Korea; 31 Neuroscience Center, Samsung Medical Center, Seoul, Republic of Korea; 32 Department of Health Sciences and Technology, Samsung Advanced Institute for Health Sciences & Technology, Sungkyunkwan University, Seoul, Republic of Korea

## Abstract

**Importance:**

The impact of late onset in myelin oligodendrocyte glycoprotein antibody–associated disease (MOGAD) is still controversial.

**Objective:**

To investigate the association of late onset MOGAD with moderate disability and relapse in Korean patients.

**Design, Setting, and Participants:**

This nationwide, multicenter, retrospective cohort study included adult patients with a diagnosis of MOGAD according to the 2023 international diagnostic criteria between August 2018 and September 2024 across 28 hospitals in South Korea.

**Exposure:**

Age at onset of MOGAD, categorized into adult-onset MOGAD (AO-MOGAD; 18-49 years) and late-onset MOGAD (LO-MOGAD; ≥50 years).

**Main Outcomes and Measures:**

The primary outcomes were time to first relapse in patients with a disease duration of 12 or more months and moderate disability, defined as Expanded Disability Status Scale (EDSS) score of 3 or greater at last follow-up.

**Results:**

A total of 350 patients (mean [SD] age at onset, 43.2 [15.0] years; 189 female [54.0%]) with a median (IQR) baseline EDSS of 3.0 (2.0-4.0) were included, with 124 patients (35.4%) with LO-MOGAD and 226 patients (64.6%) with AO-MOGAD. The LO-MOGAD group had less frequent brain involvement than the AO-MOGAD group at onset (26 patients [21.0%] vs 75 patients [33.2%]; *P* = .02) and during the disease course (28 patients [22.6%] vs 95 patients [42.0%]; *P* < .001), while optic neuritis or myelitis was comparable between the 2 groups. The LO-MOGAD group showed more frequent monophasic course (55 of 95 patients [57.9%] vs 75 of 188 patients [39.9%]; *P* = .004), but higher EDSS score at last follow-up (median [IQR], 2.0 [1.0-2.0] vs 1.0 [0.0-2.0]; *P* < .001) compared with those in the AO-MOGAD group. However, late onset was not significantly associated with the time to first relapse in multivariable analysis (adjusted hazard ratio, 0.72; 95% CI, 0.48-1.08; *P* = .11), which was consistent after propensity score matching. By contrast, late onset was associated with a significantly higher risk of moderate disability at the last follow-up (adjusted odds ratio, 2.84; 95% CI, 1.39-5.80; *P* = .004).

**Conclusions and Relevance:**

In this cohort study of MOGAD, late onset was not associated with a risk of relapse but with a higher risk of moderate disability at follow-up. Prospective studies with longer follow-up periods are warranted to better understand and manage patients with late-onset disease.

## Key Points

**Question:**

Is late-onset myelin oligodendrocyte glycoprotein antibody–associated disease (MOGAD) associated with a higher risk of time to first relapse or disability compared with adult-onset MOGAD?

**Findings:**

In this cohort study of 350 adult patients with MOGAD in South Korea, late onset was not uncommon, accounting for 35% of patients. Late onset was not associated with time to first relapse but was independently associated with a higher risk of moderate disability at last follow-up.

**Meaning:**

These findings suggest that patients with late-onset MOGAD may require closer monitoring and early disability-focused interventions.

## Teaser

This cohort study compares characteristics of adult-onset vs late-onset myelin oligodendrocyte glycoprotein (MOG) antibody–associated disease and examines associations with risk of relapse and moderate disability.

## Introduction

Myelin oligodendrocyte glycoprotein antibody–associated disease (MOGAD) is an acquired central nervous system inflammatory demyelinating disease that affects the optic nerve, spinal cord, and/or brain and is characterized by the presence of myelin oligodendrocyte glycoprotein (MOG) immunoglobulin G antibodies. Unlike other central nervous system demyelinating diseases such as multiple sclerosis (MS) or neuromyelitis optica spectrum disorder (NMOSD), MOGAD is more prevalent in children, with distinct clinical phenotypes observed in pediatric and adult patients.^1,2,3^ However, MOGAD can occur at any age with reported range of 1 to 85 years.^1,4,5,6^ As antibody testing becomes widespread and the elderly population grows globally, understanding MOGAD in older patients and establishing management strategies are crucial.

Late-onset MOGAD (LO-MOGAD) have been less explored compared with late-onset MS and NMOSD.^7,8,9^ A recent study reported that LO-MOGAD exhibited different clinical and radiological features compared with pediatric and young adult–onset patients.^10^ However, studies on long-term outcomes such as relapse and disability are still scarce and inconclusive in MOGAD.^11,12^ This is likely attributable to the rarity of MOGAD, with reported annual incidence of 3.4 to 4.8 per million and prevalence of 1.3 to 2.5 per 100 000, and to its relatively recent recognition as a distinct clinical entity compared with MS and NMOSD.^2,4^ In this study, we compared the characteristics of patients with LO-MOGAD and adult-onset MOGAD (AO-MOGAD) using a nationwide, multicenter registry in Korea and investigated the association of late onset with the risk of relapse and moderate disability.

## Methods

### Study Populations

In this nationwide, multicenter retrospective cohort study, we enrolled adult patients with MOGAD with detailed medical records between August 2018 and September 2024 from 28 secondary or tertiary hospitals in South Korea. Inclusion criteria were as follows: (1) diagnosis of MOGAD according to the 2023 international MOGAD diagnostic criteria and (2) patients with Expanded Disability Status Scale (EDSS) score assessed at least 4 weeks after a recent relapse.^4^ Patients with onset before 18 years of age (34 individuals), with anti-aquaporin-4 antibody (3 individuals), and without last EDSS data (11 individuals) were excluded. Duplicate records were merged (17 individuals). Patients were categorized by age at onset into AO-MOGAD (18-49 years) and LO-MOGAD (≥50 years) groups (eFigure 1 in Supplement 1). This study followed the Strengthening the Reporting of Observational Studies in Epidemiology (STROBE) reporting guideline and was approved by the institutional review board of the Samsung Medical Center. Written informed consent was obtained from all participants.

### Data Collection

Demographics, dates, and clinical phenotype of MOGAD onset and relapses, laboratory findings, including MOG immunoglobulin G serostatus and cerebrospinal fluid (CSF) analysis, magnetic resonance imaging (MRI) features, EDSS and visual functional system scores at onset (at the nadir) and at the last follow-up, and treatment data, including acute and preventive immunotherapies were obtained by reviewing the medical records. MRI scans of the orbit, brain, and spinal cord throughout the disease course were reviewed based on supportive criteria.^4^ Monophasic course was defined as no relapse in patients with disease duration over 12 months. The annualized relapse rate (ARR) was calculated using the total number of relapses excluding the index event.

Serum MOG immunoglobulin G assays were performed by the previously described live cell-based flow cytometry assay or the live cell-based assay, using full-length human MOG.^13,14,15^ CSF data included white blood cell count, protein levels, Immunoglobulin G index, and oligoclonal bands; pleocytosis was defined as more than10 white blood cells per microliter. Treatment history included acute treatment at onset and immunosuppressant (IS) use as a preventive treatment, including the dates of initiation and discontinuation. IS included oral steroid and nonsteroid IS, such as azathioprine, mycophenolate mofetil, rituximab, satralizumab, tocilizumab, and intravenous immunoglobulin (IVIG). Preventive treatment was defined as IS use for 3 or more months. Patients with IS for 3 or more months before the first relapse or the last follow-up (if relapse-free) were classified as preventive IS use before relapse. Treatment-duration ratio was calculated as treatment duration/overall disease duration.

### Definitions of Time to First Relapse and Moderate Disability

Relapse was defined as a new clinical attack occurring more than 30 days following onset of the previous attack.^4^ Each relapse was confirmed by the treating neurologist, with MRI support when available. Time to first relapse was defined as the interval from disease onset to the first relapse. Moderate disability at the last follow-up was defined as greater than or equal to an EDSS score of 3 at the last visit, indicating moderate disability in 1 functional system or mild disability in 3 to 4 functional systems.^16^

### Statistical Analysis

Categorical variables are reported as frequencies (percentages) and were analyzed using the χ^2^ or Fisher exact test. Continuous variables are presented as the mean (SD) or median (IQR) and were compared using the independent *t* test or Mann-Whitney *U* test, 1-way analysis of variance, or Kruskal-Wallis test. After comparing clinical characteristics of LO-MOGAD and AO-MOGAD, exploratory sensitivity analysis was performed by stratifying age into 18 to 39 years, 40 to 59 years, and 60 years or older to further examine the distribution of very late-onset cases (≥60 years). For pairwise group comparisons, *P* values were adjusted using the Bonferroni method owing to multiple comparisons.

Cox proportional hazards regression analysis identified factors associated with the time to first relapse in patients with a disease duration of 12 or more months. Covariate for preventive treatment was considered as a time dependent variable. Binary logistic regression identified factors associated with moderate disability at last follow-up. Variables with *P* < .10 in univariable analyses were included into multivariable models after assessing multicollinearity if their variance inflation factor was less than 3. The proportional hazards assumption was verified by inspecting the Schoenfeld residual plots for covariates. Variables with minimal missingness (1-2 cases) were analyzed using complete case analysis. Body mass index (BMI; calculated as weight in kilograms divided by height in meters squared), which had a substantial proportion of missing values, was excluded from the multivariable models to avoid excessive loss of sample size. Multiple imputation was not performed.

A 1:1 propensity score matching (PSM) was conducted with a caliper of 0.1, using the nearest neighbor method to balance the covariate between the LO-MOGAD and AO-MOGAD groups. Covariate balance was confirmed using standardized mean differences less than 0.2 (eFigure 2 in Supplement 1).^17,18^ Time to first relapse was analyzed in patients with a disease duration of 12 or more months and the propensity scores were estimated using logistic regression, with covariates including sex, BMI, onset phenotype (optic neuritis, myelitis, and brain), EDSS score at onset, acute treatment at onset, and preventive treatment before relapse. For disability risk analysis, the propensity score was estimated with covariates including sex, BMI, disease duration, onset phenotype (optic neuritis, myelitis, and brain), EDSS score at onset, and acute treatment at onset. To account for the matched design, a Cox proportional hazards model with a robust variance estimator was used to assess the association of LO-MOGAD with time to first relapse and a generalized estimating equation was used to compare moderate disability between the groups. The relapse-free survival curve was estimated using the Kaplan-Meier method.

In 115 patients with disease duration of 48 months or longer, ARR and total number of attacks were plotted at 6-month intervals. A generalized estimating equation with a negative binomial distribution was used to account for repeated measures and overdispersion in the number of attacks. *P* < .05 was considered statistically significant. All statistical analyses were performed using R software version 4.3.1 (R Project for Statistical Computing) and SPSS Statistics version 29.0. (SPSS Inc).

## Results

### Clinical and Demographic Data

A total of 350 patients (mean [SD] age at onset, 43.2 [15.0] years; 189 female [54.0%]; 50 [14.2%] developing MOGAD at ≥60 years) with a median (IQR) baseline EDSS of 3.0 (2.0-4.0) were included, with 124 patients (35.4%) in the LO-MOGAD group and 226 patients (64.6%) in the AO-MOGAD group (Table 1). The sex ratio and BMI were not significantly different between the 2 groups. Optic neuritis was the most common phenotype in both groups, followed by myelitis. Brain involvement was less frequent in the LO-MOGAD group at onset (26 patients [21.0%] vs 75 patients [33.2%]; *P* = .02) and during the entire disease course (28 patients [22.6%] vs 95 patients [42.0%]; *P* < .001). Compared with the AO-MOGAD group, the LO-MOGAD group showed more frequent monophasic course (55 of 95 patients [57.9%] vs 75 of 188 patients [39.9%]; *P* = .004), fewer total attacks (median [IQR], 1.0 [1.0-2.0] vs 1.5 [1.0-3.0]; *P* < .001), lower ARR (median [IQR], 0.00 [0.00-0.38] vs 0.14 [0.00-0.39]; *P* = .02), and shorter disease duration (median [IQR], 26.5 [9.4-44.1] vs 35.1 [14.9-82.2] months; *P* < .001). Patients with LO-MOGAD had higher EDSS (median [IQR], 2.0 [1.0-2.0] vs 1.0 [0.0-2.0]; *P* < .001) and visual functional system (median [IQR], 1.0 [0.0-2.0] vs 0.0 [0.0-1.0]; *P* = .03) scores at last follow-up than patients with AO-MOGAD, although EDSS and visual functional system scores at onset were not different between the 2 groups. Overall, 53 patients (15.1%) reached moderate disability (EDSS ≥3) at last follow-up. CSF pleocytosis was less frequent in the LO-MOGAD group (21 of 94 patients [22.3%] vs 73 of 177 patients [41.2%]; *P* = .002). Characteristic brain MRI findings for MOGAD, such as multiple ill-defined T2 lesions in supratentorial and infratentorial white matter; ill-defined T2 lesions involving pons, middle cerebellar peduncle or medulla; and cortical lesions with or without lesional and overlying meningeal enhancement, were significantly less frequent in the LO-MOGAD than in the AO-MOGAD group (Table 1). Acute treatment at onset did not differ between the LO-MOGAD and AO-MOGAD groups; more than 80% of the patients received IV steroids (284 patients [81.8%]) with or without additional IVIG or plasmapheresis. Preventive treatment was less frequent in the LO-MOGAD group than in the AO-MOGAD group; nonsteroid IS use was less frequent in the LO-MOGAD group than in the AO-MOGAD group (66 of 124 patients [53.2%] vs 151 of 225 patients [67.1%]; *P* = .01), whereas there was no significant difference in the treatment with oral steroids. Particularly, rituximab (4 patients [3.2%] vs 22 patients [9.7%]; *P* = .03) and IVIG (2 patients [1.6%] vs 19 patients [8.4%]; *P* = .01) use were significantly more frequently observed in AO-MOGAD group. No one in LO-MOGAD group was treated with satralizumab or tocilizumab. However, the treatment-duration ratio of IS and nonsteroid IS did not differ between the 2 groups. Regarding adverse effects of nonsteroid IS, no significant differences in adverse effect frequencies were observed between the LO-MOGAD and AO-MOGAD across all studied nonsteroid ISs. Details are provided in eTable 1 in Supplement 1.

**Table 1.** Clinical Characteristics of Patients With Late-Onset and Adult-Onset MOGAD^a^

Characteristic	Participants, No./total No. (%)			*P* value
	Late-onset MOGAD (n = 124)	Adult-onset MOGAD (n = 226)	All MOGAD (N = 350)	
Age at onset, mean (SD), y	60.1 (7.1)	34.0 (8.7)	43.2 (15.0)	<.001
Sex				
Female	75/124 (60.5)	114/226 (50.4)	189/350 (54.0)	.07
Male	49/124 (39.5)	112/226 (49.5)	161/350 (46.0)	
Body mass index, mean (SD)^b^	24.2 (3.3)	24.2 (4.6)	24.2 (4.2)	.91
Disease duration, median (IQR), mo	26.5 (9.4-44.1)	35.1 (14.9-82.2)	29.9 (13.1-63.1)	<.001
Attack type at onset				
Optic neuritis	67/124 (54.0)	116/226 (51.3)	183/350 (52.3)	.63
Myelitis	35/124 (28.2)	61/226 (27.0)	96/350 (27.4)	.80
Brain^c^	26/124 (21.0)	75/226 (33.2)	101/350 (28.9)	.02
Attack type during the course				
Optic neuritis	70/124 (56.5)	143/226 (63.3)	213/350 (60.9)	.21
Myelitis	40/124 (32.3)	85/226 (37.6)	125/350 (35.7)	.32
Brain^c^	28/124 (22.6)	95/226 (42.0)	123/350 (35.1)	<.001
Relapse data				
Monophasic course	55/95 (57.9)	75/188 (39.9)	130/283 (45.9)	.004
Total No. of attacks, median (IQR)	1.0 (1.0-2.0)	1.5 (1.0-3.0)	1.0 (1.0-3.0)	<.001
Annualized relapse rate, median (IQR)^d^	0.00 (0.00-0.38)	0.14 (0.00-0.39)	0.05 (0.00-0.38)	.02
Disease disability, EDSS score				
At onset, median (IQR)	3.0 (2.0-4.0)	3.0 (2.0-4.0)	3.0 (2.0-4.0)	.94
At last visit				
Median (IQR)	2.0 (1.0-2.0)	1.0 (0.0-2.0)	1.0 (0.0-2.0)	<.001
EDSS score ≥3	25/124 (20.2)	28/226 (12.4)	53/350 (15.1)	.05
EDSS score ≥6	4/124 (3.2)	3/226 (1.3)	7/350 (2.0)	.23
Visual disability, visual functional system score, median (IQR)				
At onset	2.5 (0.0-5.0)	2.0 (0.0-5.0)	2.0 (0.0-5.0)	.45
At last follow-up	1.0 (0.0-2.0)	0.0 (0.0-1.0)	0.0 (0.0-1.0)	.03
MOG-IgG				
Low-positive MOG-IgG	18/124 (14.5)	18/226 (8.0)	36/350 (10.3)	.05
Seronegative conversion	21/80 (26.3)	42/141 (29.8)	63/221 (28.5)	.58
Cerebrospinal fluid analysis				
White blood cell count, median (IQR), cells/μL	3 (1-8)	5 (1-40)	5 (1-23)	.003
Pleocytosis	21/94 (22.3)	73/177 (41.2)	94/271 (34.7)	.002
Protein, median (IQR), mg/dL	43.5 (32.5-59.8)	40.5 (30.0-57.8)	42.0 (31.0-58.0)	.38
Oligoclonal band positive	4/87 (4.6)	17/163 (10.4)	21/250 (8.4)	.11
IgG index, median (IQR)	0.55 (0.49-0.63)	0.57 (0.49-0.68)	0.56 (0.49-0.66)	.28
Magnetic resonance imaging findings				
Optic nerve				
Bilateral involvement	28/124 (22.6)	49/226 (21.7)	77/350 (22.0)	.85
Longitudinal optic nerve involvement	44/124 (35.5)	67/226 (29.6)	111/350 (31.7)	.26
Perineural optic sheath enhancement	42/124 (33.9)	62/226 (27.4)	104/350 (29.7)	.21
Optic disc edema	34/124 (27.4)	56/226 (24.8)	90/350 (25.7)	.59
Spinal cord				
Longitudinally extensive myelitis	20/124 (16.1)	38/226 (16.8)	58/350 (16.6)	.87
Central cord lesion or H-sign	16/124 (12.9)	45/226 (19.9)	61/350 (17.4)	.10
Conus lesion	4/124 (3.2)	15/226 (6.6)	19/350 (5.4)	.18
Brain				
Multiple ill-defined T2 hyperintensity lesion in supratentorial and often infratentorial white matter	21/124 (16.9)	68/226 (30.1)	89/350 (25.4)	.007
Deep gray matter involvement	12/124 (9.7)	36/226 (15.9)	48/350 (13.7)	.10
Ill-defined T2-hyperintensity involving pons, middle cerebellar peduncle, or medulla	17/124 (13.7)	63/226 (27.9)	80/350 (22.9)	.003
Cortical lesion with or without lesional and overlying meningeal enhancement	6/124 (4.8)	30/226 (13.3)	36/350 (10.3)	.01
Acute treatment at onset				
Any treatment	102/123 (82.9)	182/224 (81.3)	284/347 (81.8)	.70
IV steroid	102/123 (82.9)	181/224 (80.8)	283/347 (81.6)	.63
IV steroid followed by IVIG or PLEX	5/123 (4.1)	19/224 (8.5)	24/347 (6.9)	.12
IV steroid and IVIG	2/123 (1.6)	11/224 (4.9)	13/347 (3.7)	.15
IV steroid and PLEX	3/123 (2.4)	10/224 (4.5)	13/347 (3.7)	.40
IVIG only	0	1/224 (0.4)	1/347 (0.3)	>.99
Preventive treatment^e^				
IS use	83/124 (66.9)	174/225 (77.3)	257/349 (73.6)	.04
Oral steroids use	24/124 (19.4)	33/226 (14.6)	57/350 (16.3)	.25
Nonsteroid IS use				
Any	66/124 (53.2)	151/225 (67.1)	217/349 (62.2)	.01
Azathioprine or mycophenolate	64/124 (51.6)	133/225 (59.1)	197/349 (56.4)	.18
Rituximab	4/124 (3.2)	22/226 (9.7)	26/350 (7.4)	.03
Satralizumab or tocilizumab	0	7/226 (3.1)	7/350 (2.0)	.05
IVIG	2/124 (1.6)	19/226 (8.4)	21/350 (6.0)	.01
Preventive treatment before relapse^f^	59/124 (47.6)	85/226 (37.6)	144/350 (41.1)	.07
Treatment-duration ratio of IS, median (IQR)^g^	0.67(0.06-0.94)	0.71(0.20-0.92)	0.70 (0.17-0.93)	.98
Treatment-duration ratio of nonsteroid IS, median (IQR)^h^	0.43 (0.00-0.90)	0.56 (0.02-0.88)	0.50 (0.00-0.89)	.16

Abbreviations: EDSS, Expanded Disability Status Scale; IS, immunosuppressant; IV, intravenous; IVIG, intravenous immunoglobulin; MOGAD, myelin oligodendrocyte glycoprotein antibody-associated disease; MOG-IgG, myelin oligodendrocyte glycoprotein-immunoglobulin G; PLEX, plasmapheresis.

^a^ Analyses were performed using the χ^2^ or Fisher exact test for categorical variables, as appropriate. The independent *t*-test or Mann-Whitney U-test was used for continuous variables, depending on the normality of the data distribution.

^b^ Body mass index was calculated as weight in kilograms divided by height in meters squared. Data include 58 missing values.

^c^ Brain included acute disseminated encephalomyelitis, cerebral monofocal or polyfocal deficits, brainstem or cerebellar deficits, and cerebral cortical encephalitis.

^d^ The evaluation of the annualized relapse rate was based on patients with a disease duration 12 months or longer (178 individuals in the adult-onset MOGAD group and 87 individuals in the late-onset MOGAD group).

^e^ Preventive treatment was defined as treatment with immunosuppressant for 3 months or longer.

^f^ Preventive treatment before relapse was defined as treatment with immunosuppressant for 3 months or longer, before relapse.

^g^ The treatment-duration ratio of IS was defined as (IS treatment duration) / (overall disease duration).

^h^ The treatment-duration-ratio of nonsteroid IS was defined as (nonsteroid IS treatment duration) / (overall disease duration).

Sensitivity analysis demonstrated that patients with very late onset (≥60 years), who accounted for 14.2% of the cohort (50 of 350 patients), showed similar differences in clinical characteristics compared with the other age groups (eTable 2 in Supplement 1). A comparison of baseline characteristics of LO-MOGAD and AO-MOGAD in patients with a disease duration of 12 or more months is presented in eTable 3 in Supplement 1.

### Factors Associated With Time to First Relapse

In univariable analysis, late onset, myelitis at onset, and preventive treatment before relapse were associated with lower risk of time to first relapse, while optic neuritis at onset was associated with higher risk of time to first relapse. In multivariable analysis adjusting for optic neuritis at onset and preventive treatment before relapse, late onset was not significantly associated with time to first relapse (adjusted hazard ratio [aHR], 0.72; 95% CI, 0.48-1.08; *P* = .11), while preventive treatment remained protective (aHR, 0.19; 95% CI, 0.11-0.33; *P* < .001) (Table 2). These findings remained consistent even when myelitis at onset was used as a covariate instead of optic neuritis (eTable 4 in Supplement 1).

**Table 2.** Cox Proportional Hazard Regression Analysis for Factors Associated With Relapses in Patients With Myelin Oligodendrocyte Glycoprotein Antibody-Associated Disease^a^

Variable	Risk of relapse					
	Unadjusted HR (95% CI)	*P* value	Model 1, adjusted HR (95% CI)	*P* value	Model 2, adjusted HR (95% CI)	*P* value
Female sex	0.76 (0.54-1.07)	.12	NA	NA	NA	NA
Late-onset	0.63 (0.42-0.94)	.02	0.64 (0.43-0.95)	.03	0.72 (0.48-1.08)	.11
Body mass index^b^	1.05 (1.00-1.11)	.05	NA	NA	NA	NA
Onset phenotype						
Optic neuritis	1.50 (1.06-2.11)	.02	1.48 (1.05-2.09)	.03	1.45 (1.03-2.05)	.04
Myelitis	0.52 (0.34-0.79)	.002	NA	NA	NA	NA
Brain^c^	1.16 (0.79-1.70)	.44	NA	NA	NA	NA
EDSS score ≥3 at onset	0.77 (0.52-1.14)	.19	NA	NA	NA	NA
Acute treatment at onset	1.02 (0.66-1.57)	.94	NA	NA	NA	NA
Preventive treatment before relapse^d^^,^^e^	0.18 (0.10-0.31)	<.001	NA	NA	0.19 (0.11-0.33)	<.001

Abbreviations: EDSS, expanded disability status scale; HR, hazard ratio; NA, not applicable.

^a^ Unadjusted HRs for univariable analysis and adjusted HRs for multivariable analysis are presented. Model 1 was adjusted for optic neuritis at onset. Model 2 was additionally adjusted for preventive treatment before relapse.

^b^ Body mass index data were excluded in multivariable analysis due to 55 missing values.

^c^ Brain included acute disseminated encephalomyelitis, cerebral monofocal or polyfocal deficits, brainstem or cerebellar deficits, and cerebral cortical encephalitis.

^d^ Preventive treatment before relapse was defined as treatment with immunosuppressant for 3 months or longer, before relapse and was treated as time-dependent covariate.

^e^ Preventive treatment before relapse had 1 missing value.

### Factors Associated With Moderate Disability at Last Follow-Up

**Figure 1.**
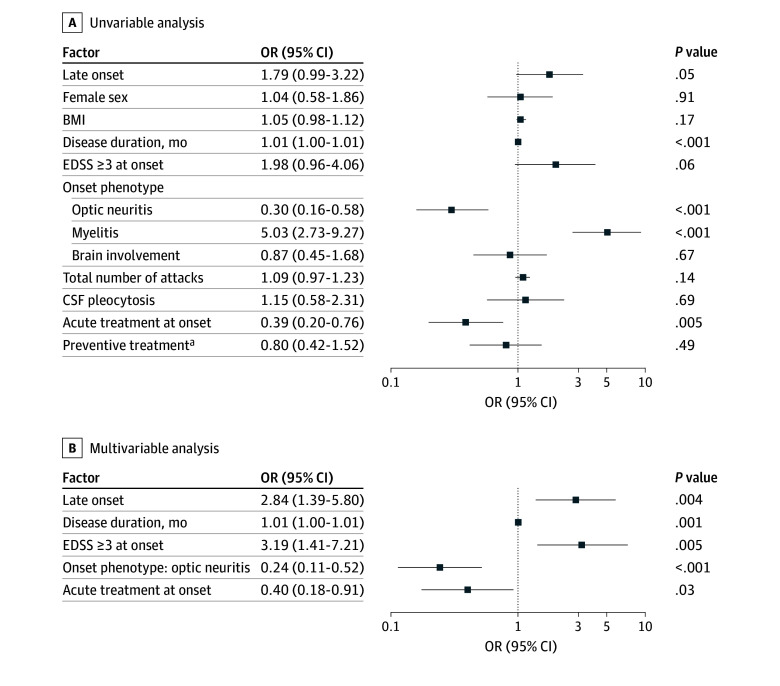
Binary Logistic Regression Analysis for Factors Associated With Moderate Disability at Last Follow-Up Unadjusted odds ratios (ORs) for univariable analysis and adjusted ORs for multivariable analysis are presented. There were 35 missing values for Expanded Disability Status Scale (EDSS) score of 3 or greater at onset and 3 missing values for acute treatments at onset. BMI indicates body mass index; CSF, cerebrospinal fluid. ^a^Preventive treatment was defined as treatment with immunosuppressant for 3 months or longer.

In univariable analysis, late onset, longer disease duration, and myelitis at onset were associated with higher risk of moderate disability at the last follow-up, while optic neuritis onset and acute treatment at onset were protective. After adjusting for disease duration, EDSS score of 3 or greater at onset, optic neuritis at onset, and acute treatment at onset, patients with LO-MOGAD demonstrated a 2.8-fold higher risk of moderate disability compared with patients with AO-MOGAD (adjusted odds ratio [aOR], 2.84; 95% CI, 1.39-5.80; *P* = .004) (Figure 1). Late onset was consistently associated with a higher risk of moderate disability when myelitis at onset was included as a covariate instead of optic neuritis (eTable 5 in Supplement 1).

### PSM

**Figure 2.**
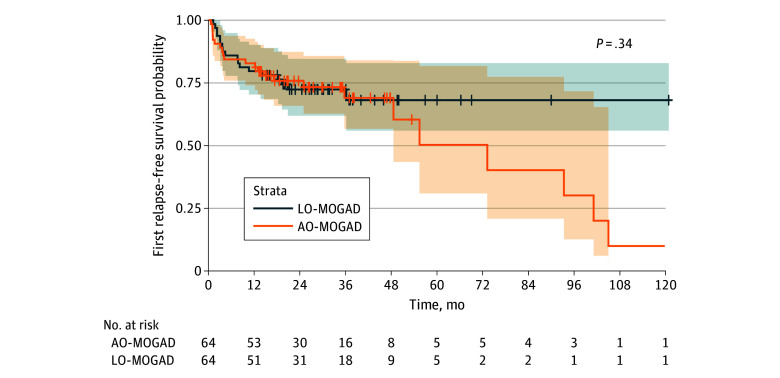
Time to First Relapse in Patients With Myelin Oligodendrocyte Glycoprotein Antibody–Associated Disease (MOGAD) After Propensity Score Matching The first relapse-free survival probability was estimated using the Kaplan-Meier method based on matched samples (64 individuals in each group). Propensity score matching was performed to balance relevant baseline characteristics between the groups. The shaded areas represent 95% CIs. The number of patients at risk at each time point is shown below. To account for the matched design, the association of late-onset disease with relapse-free survival was assessed using a Cox proportional hazards model with a robust variance estimator. AO-MOGAD indicates adult-onset MOGAD; LO-MOGAD, late-onset MOGAD.

In the analysis of time to first relapse in patients with a disease duration 12 or more months (64 patients in each group), frequency of monophasic course, total number of attacks, and ARR were comparable between LO-MOGAD and AO-MOGAD (eTable 6 in Supplement 1). There was no significant difference in time to first relapse between the LO-MOGAD and AO-MOGAD groups (aHR, 0.99; 95% CI, 0.96-1.02, *P* = .34) (Figure 2). For moderate disability, 101 patients were included in each group after PSM. Patients with LO-MOGAD had a higher risk of moderate disability at the last follow-up (aOR, 2.53; 95% CI, 1.09-5.88; *P* = .03). This finding was consistent after additional adjustment for covariates with standardized mean differences greater than 0.1, including sex, BMI, and EDSS score at onset for relapse risk (aHR, 0.79; 95% CI, 0.45-1.37; *P* = .40) and disease duration, and EDSS score at onset for disability risk (aOR, 2.57; 95% CI, 1.14-5.80; *P* = .02).

### ARR

**Figure 3.**
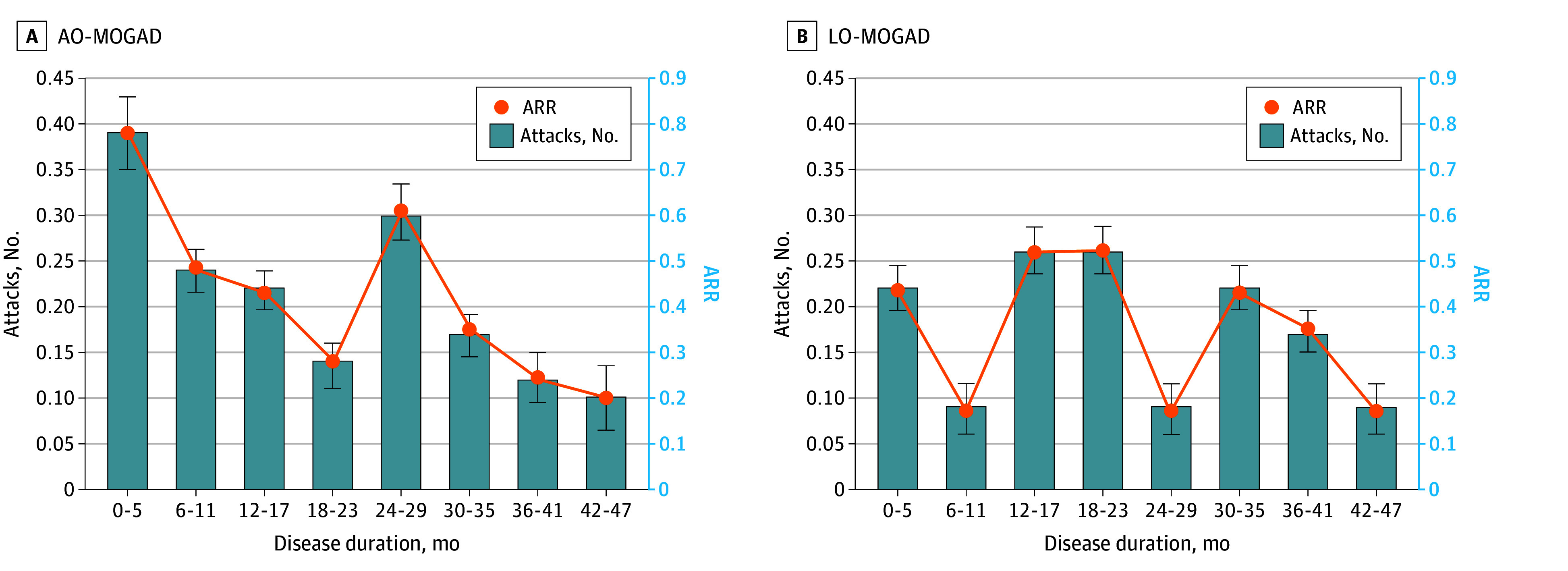
Longitudinal Patterns of Relapse Activity Over 48 Months Among Those With Adult-Onset (AO) and Late-Onset (LO) Myelin Oligodendrocyte Glycoprotein Antibody–Associated Disease (MOGAD) The number of attacks and annualized relapse rate (ARR) were assessed at 6-month intervals among patients with disease duration of 48 months or longer. The number of attacks and ARR are shown for each interval as the mean and standard deviation (error bar). A generalized estimating equation with a negative binomial distribution was used to account for repeated measures and overdispersion. The longitudinal ARR patterns over time did not differ significantly between the 2 groups (*P* = .25).

We analyzed 115 patients with a disease duration of at least 4 years, including 92 patients with AO-MOGAD and 23 patients with LO-MOGAD, and calculated the ARR at 6-month intervals over a 4-year period (Figure 3). There was no significant difference in the longitudinal ARR changes between the 2 groups. Overall, the ARR remained less than 1.0 in both the LO-MOGAD and AO-MOGAD groups throughout the 4-year study period.

## Discussion

Our nationwide cohort study in South Korea demonstrated that LO-MOGAD is not uncommon and has several distinct features compared with AO-MOGAD. While late onset was not associated with a risk of relapse, it was independently associated with a higher risk of disability.

One-third of patients in our adult MOGAD registry was classified as LO-MOGAD, a higher proportion than in prior reports, even when comparing only adult patients.^1,10,11^ Notably, 14.2% (50 of 350 adult patients) developed MOGAD at age 60 years or older, which surpasses the 6.0% (16 of 268 adult patients) reported in a French MOGAD cohort.^19^ These proportions are comparable to late-onset NMOSD, where late onset cases (age ≥50 years) represent 29% to 45%,^20,21,22^ but are higher than MS, in which only 5% to 12% are late-onset.^23,24^ In MS, the age at onset has increased in recent decades, and this may be attributable to the aging of the population, improved diagnostic methods, or changes in diagnostic criteria.^25,26^ More widespread and feasible antibody testing, increased awareness of disease, and population aging in South Korea may account for the higher LO-MOGAD proportion in our cohort.

LO-MOGAD was associated with less frequent brain involvement and less CSF pleocytosis, which may reflect a less inflammatory disease course in older patients. In MS, aging shifts the disease activity from a relapsing inflammatory course toward progressive neurodegeneration with fewer active demyelinating lesions observed in older patients and in those with longer disease duration.^7,27,28^ In NMOSD, older age at onset correlates with lower ARR.^21,29^ In our cohort, the monophasic course was more frequent and ARR was lower in LO-MOGAD compared with AO-MOGAD. However, these differences were no longer significant after PSM, indicating that a reduced inflammatory activity was largely attributable to other baseline differences rather than age at onset in MOGAD. This finding was supported by the multivariable Cox and PSM analyses showing that late onset was not associated with time to first relapse. Consistently, a French cohort found that only maintenance treatment after onset was associated with relapse, while other clinical factors including age were not.^30^ Recently, in the comparison of patients with early and late adult–onset MOGAD, there was no difference in relapse risk, although this was not confirmed after PSM.^12^ By contrast, a Chinese study suggested lower relapse risk in patients with LO-MOGAD, but they had much lower maintenance therapy rates compared with ours (33% vs 67%).^11^ In our study, the association of late onset with relapse disappeared after adjusting for preventive treatment, underscoring the dominant effect of treatment emphasized in prior studies.^11,30,31,32^

Another notable finding in our cohort was that patients with LO-MOGAD were less likely to receive IS, particularly, monoclonals compared with AO-MOGAD. Moreover, oral nonsteroid IS was most frequently used regardless of age group, despite emerging evidences supporting the benefits of immunotherapies such as IVIG or tocilizumab.^33,34^ This may be attributed to high costs due to the lack of reimbursement in Korea and as well as the fact that our cohort data encompasses a period prior to recommendations of these treatments. Still, prospective clinical trials are essential to establish standardized guidelines for relapse prevention in patients with LO-MOGAD and AO-MOGAD, considering the retrospective design in previous studies.

Overall, 15% of our patients reached moderate disability (EDSS score ≥3) at the last follow-up. Notably, patients with LO-MOGAD showed higher residual disability despite similar baseline EDSS score and shorter disease duration. These findings align with previous studies reporting increased disability risks in late onset patients with MOGAD and NMOSD.^11,35^ While another study found no difference in last EDSS scores between early-onset MOGAD and LO-MOGAD, this study did not conduct PSM analysis and the authors acknowledged that residual disability defined as EDSS of 2 or greater could be confounded by neurologic abnormalities normally observed with aging.^12^

Although the discrepancy between fewer relapses and higher disability in LO-MOGAD could raise the question of progression independent of relapse activity, recent studies have consistently shown that progression independent of relapse activity is rare in MOGAD and that disability is primarily relapse-associated.^36,37^ The greater disability burden observed in LO-MOGAD is therefore likely multifactorial, reflecting incomplete recovery from the first attack in the context of biological aging processes—such as reduced neuroplasticity and remyelination capacity—as well as age-related factors including frailty and comorbidities. With aging, neuroplasticity and regenerative capacity decline, especially the central nervous system ability to remyelinate after demyelinating attacks.^28,38,39^ Similarly in MS, older age is associated with limited recovery from relapses, and late-onset disease shows faster disability accumulation.^23,40^ Late-onset NMOSD also often shows worse prognosis, characterized by more severe motor disability as well as age-related comorbidities.^9,41^ Their outcomes are particularly poor in patients aged 70 years or older, with longer cord lesions and reduced resilience to relapses contributing to disability accumulation.^22,42^

### Limitations

Our study had several limitations. First, missing data were unavoidable due to the retrospective design; however, potential confounding was mitigated through PSM. Second, the shorter disease duration in LO-MOGAD may have underestimated the long-term disease burden. Third, late onset was defined using an arbitrary cutoff of 50 years, although this cutoff has been widely used in MS and NMOSD, and a prior MOGAD study found no difference between patients aged 50 to 59 years and 60 years or older.^12^ Fourth, the retrospective design precluded a detailed assessment of complex regimens, including concomitant corticosteroid use with nonsteroid IS (eg, as bridging therapy); exact overlap durations and dosages could not be quantified. Fifth, baseline frailty and comorbidities, potential confounders of disability outcomes, were not systematically evaluated.

## Conclusions

In this cohort study of MOGAD, late onset was associated with a higher risk of moderate disability but was not associated with the risk of relapse, which was mainly attributable to preventive treatment and onset phenotypes. Prospective studies with longer follow-up are warranted to validate these findings and to guide treatment in late-onset patients.

## Notes

Supplement 1. eFigure 1. Flow Chart of the Study Population eFigure 2. Covariate Balance and Propensity Score Distribution eTable 1. Adverse Effects of Non-Steroid Immunosuppressant in Patients with Myelin Oligodendrocyte Glycoprotein Antibody-Associated Disease eTable 2. Clinical Characteristics of Patients With Myelin Oligodendrocyte Glycoprotein Antibody-Associated Disease According to the Age Groups eTable 3. Baseline Characteristics of Patients With LO-MOGAD and AO-MOGAD With Disease Duration ≥12 Months eTable 4. Cox Proportional Hazard Regression Analysis for Factors Associated With Relapses in Patients with MOGAD Using Myelitis Onset as a Covariate eTable 5. Binary Logistic Regression Analysis for Factors Associated With Moderate Disability at Last Follow-Up Using Myelitis Onset as a Covariate eTable 6. Relapse Outcomes in Lat-Onset and Adult-Onset Myelin Oligodendrocyte Glycoprotein Antibody-Associated Disease After Propensity Score Matching

Supplement 2. Data Sharing Statement

## References

[1] Brill L, Ganelin-Cohen E, Dabby R, . Age-related clinical presentation of MOG-IgG seropositivity in Israel. Front Neurol. 2021;11:612304. doi:10.3389/fneur.2020.612304 33584514 PMC7874097

[2] Hor JY, Fujihara K. Epidemiology of myelin oligodendrocyte glycoprotein antibody-associated disease: a review of prevalence and incidence worldwide. Front Neurol. 2023;14:1260358. doi:10.3389/fneur.2023.1260358 37789888 PMC10542411

[3] Sechi E, Cacciaguerra L, Chen JJ, . Myelin oligodendrocyte glycoprotein antibody-associated disease (MOGAD): a review of clinical and MRI features, diagnosis, and management. Front Neurol. 2022;13:885218. doi:10.3389/fneur.2022.885218 35785363 PMC9247462

[4] Banwell B, Bennett JL, Marignier R, . Diagnosis of myelin oligodendrocyte glycoprotein antibody-associated disease: international MOGAD panel proposed criteria. Lancet Neurol. 2023;22(3):268-282. doi:10.1016/S1474-4422(22)00431-8 36706773

[5] Jurynczyk M, Messina S, Woodhall MR, . Clinical presentation and prognosis in MOG-antibody disease: a UK study. Brain. 2017;140(12):3128-3138. doi:10.1093/brain/awx276 29136091

[6] Kaneko K, Sato D, Takahashi T, . Clinical, MRI and laboratory features of myelin oligodendrocyte glycoprotein (MOG)-antibody-associated neurologic disease: a study of 259 cases. Mult Scler J. 2017;23(3)(suppl_3):286. doi:10.1177/1352458517731404

[7] Knowles S, Middleton R, Cooze B, ; UK MS Register Research Group. Comparing the pathology, clinical, and demographic characteristics of younger and older-onset multiple sclerosis. Ann Neurol. 2024;95(3):471-486. doi:10.1002/ana.26843 38061895

[8] Buscarinu MC, Reniè R, Morena E, . Late-onset MS: disease course and safety-efficacy of DMTS. Front Neurol. 2022;13:829331. doi:10.3389/fneur.2022.829331 35356454 PMC8960027

[9] Seok JM, Cho HJ, Ahn SW, . Clinical characteristics of late-onset neuromyelitis optica spectrum disorder: a multicenter retrospective study in Korea. Mult Scler. 2017;23(13):1748-1756. doi:10.1177/1352458516685416 28058965

[10] Huang Y, Luo W, Cheng X, . Clinical and imaging features of patients with late-onset myelin oligodendrocyte glycoprotein antibody-associated disease. Mult Scler Relat Disord. 2024;82:105405. doi:10.1016/j.msard.2023.105405 38194895

[11] Fan Y, Wang Z, Wu Y, . Fewer relapses and worse outcomes of patients with late-onset myelin oligodendrocyte glycoprotein antibody-associated disease. J Neurol Neurosurg Psychiatry. 2025;96(7):655-664. doi:10.1136/jnnp-2024-334613 39643428

[12] Dinoto A, Cacciaguerra L, Vorasoot N, . Clinical features and factors associated with outcome in late adult-onset myelin oligodendrocyte glycoprotein antibody-associated disease. Neurology. 2025;104(10):e213557. doi:10.1212/WNL.0000000000213557 40324120 PMC12042097

[13] Waters P, Woodhall M, O’Connor KC, . MOG cell-based assay detects non-MS patients with inflammatory neurologic disease. Neurol Neuroimmunol Neuroinflamm. 2015;2(3):e89. doi:10.1212/NXI.0000000000000089 25821844 PMC4370386

[14] Kwon YN, Kim B, Kim JS, . Myelin oligodendrocyte glycoprotein-immunoglobulin G in the CSF: clinical implication of testing and association with disability. Neurol Neuroimmunol Neuroinflamm. 2021;9(1):e1095. doi:10.1212/NXI.0000000000001095 34711644 PMC8554713

[15] Seok JM, Waters P, Jeon MY, . Clinical usefulness of a cell-based assay for detecting myelin oligodendrocyte glycoprotein antibodies in central nervous system inflammatory disorders. Ann Lab Med. 2024;44(1):56-63. doi:10.3343/alm.2024.44.1.56 37665286 PMC10485852

[16] Kurtzke JF. Rating neurologic impairment in multiple sclerosis: an expanded disability status scale (EDSS). Neurology. 1983;33(11):1444-1452. doi:10.1212/WNL.33.11.1444 6685237

[17] Cohen J. Statistical Power Analysis for the Behavioral Sciences. 2nd ed. Routledge; 2013. doi:10.4324/9780203771587

[18] McCaffrey DF, Griffin BA, Almirall D, Slaughter ME, Ramchand R, Burgette LF. A tutorial on propensity score estimation for multiple treatments using generalized boosted models. Stat Med. 2013;32(19):3388-3414. doi:10.1002/sim.5753 23508673 PMC3710547

[19] Cobo-Calvo A, Ruiz A, Rollot F, ; NOMADMUS, KidBioSEP, and OFSEP study groups. Clinical Features and risk of relapse in children and adults with myelin oligodendrocyte glycoprotein antibody-associated disease. Ann Neurol. 2021;89(1):30-41. doi:10.1002/ana.25909 32959427

[20] Min W, Zhang L, Wang S, Xue M, Guo C, Zhu M. Clinical characteristics of late-onset neuromyelitis optica spectrum disorder. Mult Scler Relat Disord. 2023;70:104517. doi:10.1016/j.msard.2023.104517 36708681

[21] Hu Y, Sun Q, Yi F, . Age of onset correlates with clinical characteristics and prognostic outcomes in neuromyelitis optica spectrum disorder. Front Immunol. 2022;13:1056944. doi:10.3389/fimmu.2022.1056944 36569880 PMC9772011

[22] Sepulveda M, Delgado-García G, Blanco Y, . Late-onset neuromyelitis optica spectrum disorder: the importance of autoantibody serostatus. Neurol Neuroimmunol Neuroinflamm. 2019;6(6):e607. doi:10.1212/NXI.0000000000000607 31471461 PMC6745725

[23] Mouresan EF, Mentesidou E, Berglund A, McKay KA, Hillert J, Iacobaeus E. Clinical Characteristics and long-term outcomes of late-onset multiple sclerosis: a Swedish nationwide study. Neurology. 2024;102(6):e208051. doi:10.1212/WNL.0000000000208051 38394472 PMC11033980

[24] Naseri A, Nasiri E, Sahraian MA, Daneshvar S, Talebi M. Clinical features of late-onset multiple sclerosis: a systematic review and meta-analysis. Mult Scler Relat Disord. 2021;50:102816. doi:10.1016/j.msard.2021.102816 33571792

[25] Habbestad A, Willumsen JS, Aarseth JH, . Increasing age of multiple sclerosis onset from 1920 to 2022: a population-based study. J Neurol. 2024;271(4):1610-1617. doi:10.1007/s00415-023-12047-9 38097800 PMC10973050

[26] Romero-Pinel L, Bau L, Matas E, . The age at onset of relapsing-remitting multiple sclerosis has increased over the last five decades. Mult Scler Relat Disord. 2022;68:104103. doi:10.1016/j.msard.2022.104103 36029708

[27] Frischer JM, Weigand SD, Guo Y, . Clinical and pathological insights into the dynamic nature of the white matter multiple sclerosis plaque. Ann Neurol. 2015;78(5):710-721. doi:10.1002/ana.24497 26239536 PMC4623970

[28] Graves JS, Krysko KM, Hua LH, Absinta M, Franklin RJM, Segal BM. Ageing and multiple sclerosis. Lancet Neurol. 2023;22(1):66-77. doi:10.1016/S1474-4422(22)00184-3 36216015

[29] Wang L, Tan H, Huang W, . Late onset neuromyelitis optica spectrum disorder with anti-aquaporin 4 and anti-myelin oligodendrocyte glycoprotein antibodies. Eur J Neurol. 2022;29(4):1128-1135. doi:10.1111/ene.15239 34967093

[30] Deschamps R, Guillaume J, Ciron J, ; as the NOMADMUS study group. Early maintenance treatment initiation and relapse risk mitigation after a first event of MOGAD in adults: the MOGADOR2 study. Neurology. 2024;103(3):e209624. doi:10.1212/WNL.0000000000209624 38991174

[31] Huda S, Whittam D, Jackson R, . Predictors of relapse in MOG antibody associated disease: a cohort study. BMJ Open. 2021;11(11):e055392. doi:10.1136/bmjopen-2021-055392 34848526 PMC8634280

[32] Molazadeh N, Bilodeau PA, Salky R, . Predictors of relapsing disease course following index event in myelin oligodendrocyte glycoprotein antibody-associated disease (MOGAD). J Neurol Sci. 2024;458:122909. doi:10.1016/j.jns.2024.122909 38335710

[33] Kang YR, Kim KH, Hyun JW, Kim SH, Kim HJ. Efficacy of tocilizumab in highly relapsing MOGAD with an inadequate response to intravenous immunoglobulin therapy: a case series. Mult Scler Relat Disord. 2024;91:105859. doi:10.1016/j.msard.2024.105859 39236649

[34] Bilodeau PA, Vishnevetsky A, Molazadeh N, . Effectiveness of immunotherapies in relapsing myelin oligodendrocyte glycoprotein antibody-associated disease. Mult Scler. 2024;30(3):357-368. doi:10.1177/13524585241226830 38314479

[35] Duchow A, Bellmann-Strobl J, Friede T, ; Neuromyelitis Optica Study Group (NEMOS). Time to disability milestones and annualized relapse rates in NMOSD and MOGAD. Ann Neurol. 2024;95(4):720-732. doi:10.1002/ana.26858 38086777

[36] Akaishi T, Misu T, Takahashi T, . Progression pattern of neurological disability with respect to clinical attacks in anti-MOG antibody-associated disorders. J Neuroimmunol. 2021;351:577467. doi:10.1016/j.jneuroim.2020.577467 33388541

[37] Camera V, Messina S, Tamanti A, . Investigating the presence of neurodegeneration independent of relapses in MOGAD compared to relapsing-remitting multiple sclerosis. Neurol Open Access. 2025;1(2):e000013. doi:10.1212/WN9.0000000000000013

[38] Perdaens O, van Pesch V. Molecular mechanisms of immunosenescene and inflammaging: relevance to the immunopathogenesis and treatment of multiple sclerosis. Front Neurol. 2022;12:811518. doi:10.3389/fneur.2021.811518 35281989 PMC8913495

[39] Filippi M, Preziosa P, Barkhof F, . The ageing central nervous system in multiple sclerosis: the imaging perspective. Brain. 2024;147(11):3665-3680. doi:10.1093/brain/awae251 39045667 PMC11531849

[40] Leone MA, Bonissoni S, Collimedaglia L, . Factors predicting incomplete recovery from relapses in multiple sclerosis: a prospective study. Mult Scler. 2008;14(4):485-493. doi:10.1177/1352458507084650 18208889

[41] Carnero Contentti E, Daccach Marques V, Soto de Castillo I, . Clinical features and prognosis of late-onset neuromyelitis optica spectrum disorders in a Latin American cohort. J Neurol. 2020;267(5):1260-1268. doi:10.1007/s00415-020-09699-2 31932911

[42] Nakahara K, Nakane S, Nagaishi A, Narita T, Matsuo H, Ando Y. Very late onset neuromyelitis optica spectrum disorders. Eur J Neurol. 2021;28(8):2574-2581. doi:10.1111/ene.14901 33960076

