# Supplementary material for: Disability and Relapse Risk in Late-Onset Myelin Oligodendrocyte Glycoprotein Antibody–Associated Disease
Source: JAMA Netw Open. 2026 Feb 13;9(2):e2559471. doi: 10.1001/jamanetworkopen.2025.59471 (PMC12905660; doi:10.1001/jamanetworkopen.2025.59471)
Supplement: Supplement 1. — eFigure 1. Flow Chart of the Study Population eFigure 2. Covariate Balance and Propensity Score Distribution eTable 1. Adverse Effects of Non-Steroid Immunosuppressant in Patients with Myelin Oligodendrocyte Glycoprotein Antibody-Associated Disease eTable 2. Clinical Characteristics of Patients With Myelin Oligodendrocyte Glycoprotein Antibody-Associated Disease According to the Age Groups eTable 3. Baseline Characteristics of Patients With LO-MOGAD and AO-MOGAD With Disease Duration ≥12 Months eTable 4. Cox Proportional Hazard Regression Analysis for Factors Associated With Relapses in Patients with MOGAD Using Myelitis Onset as a Covariate eTable 5. Binary Logistic Regression Analysis for Factors Associated With Moderate Disability at Last Follow-Up Using Myelitis Onset as a Covariate eTable 6. Relapse Outcomes in Lat-Onset and Adult-Onset Myelin Oligodendrocyte Glycoprotein Antibody-Associated Disease After Propensity Score Matching [file jamanetwopen-e2559471-s001.pdf]

Supplemental Online Content

Ju H, Kim KH, Woo SY, et al. Disability and relapse risk in late-onset myelin oligodendrocyte glycoprote in antibody–associated disease. *JAMA Netw. Open*. 2026;9(2):e2559471. doi:10.1001/jamanetworkopen.2025.59471

eFigure 1. Flow Chart of the Study Population

eFigure 2. Covariate Balance and Propensity Score Distribution

eTable 1 Adverse Effects of Non-Steroid Immunosuppressant in Patients with Myelin Oligodendrocyte Glycoprotein Antibody-Associated Disease

eTable 2. Clinical Characteristics of Patients With Myelin Oligodendrocyte Glycoprotein Antibody-Associated Disease According to the Age Groups

eTable 3. Baseline Characteristics of Patients With LO-MOGAD and AO-MOGAD With Disease Duration ≥12 Months

eTable 4. Cox Proportional Hazard Regression Analysis for Factors Associated With Relapses in Patients with MOGAD Using Myelitis Onset as a Covariate

eTable 5. Binary Logistic Regression Analysis for Factors Associated With Moderate Disability at Last Follow-Up Using Myelitis Onset as a Covariate

eTable 6. Relapse Outcomes in Lat-Onset and Adult-Onset Myelin Oligodendrocyte Glycoprotein Antibody-Associated Disease After Propensity Score Matching

This supplemental material has been provided by the authors to give readers additional information about their work.

© 2026 Ju H et al. *JAMA Network Open*.

eFigure 1. Flow chart of the study population

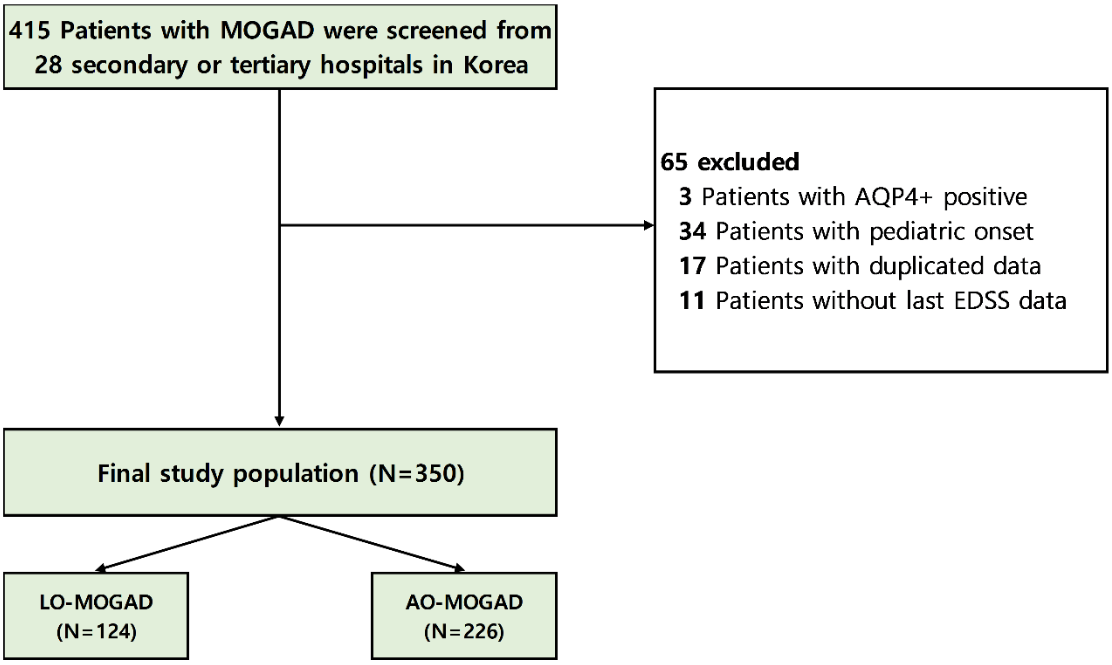

© 2026 Ju H et al. *JAMA Network Open*.

eFigure 2. Covariate Balance and Propensity Score Distribution

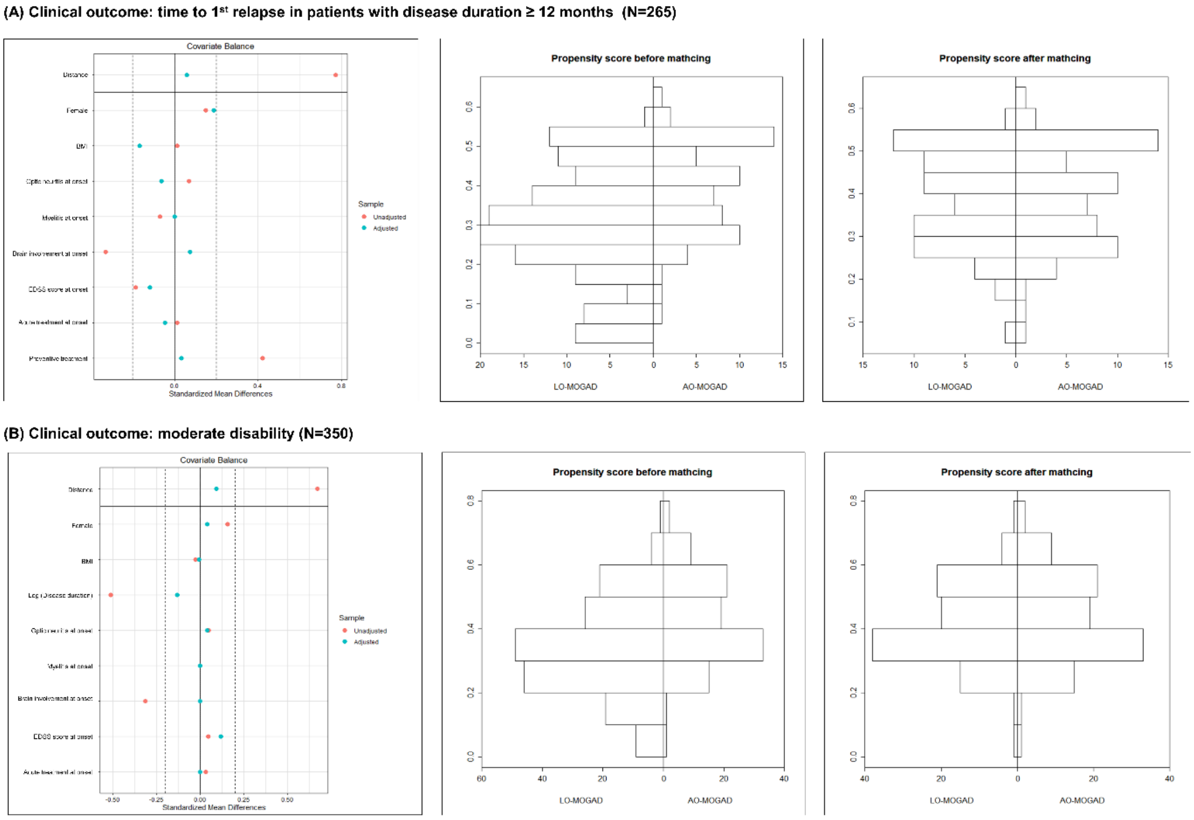

(A) For relapse risk analysis, the propensity score was estimated before relapse for the patients with disease duration ≥12months using logistic regression with covariates including sex (female), body mass index (BMI), onset phenotype (optic neuritis, myelitis, and brain), EDSS score at onset, acute treatment at onset, and preventive treatment.
Covariate balance and propensity score distribution for the clinical outcome of time to first relapse among patients with disease duration ≥ 12 months (N= 265). A total of 64 late-onset MOGAD (LO-MOGAD) patients were matched to 64 adult-onset MOGAD (AO-MOGAD) patients. The left panel shows the standardized mean differences (SMDs) of covariates before and after matching, with the dotted line indicating the acceptable threshold (SMD= ±0.2). The middle and right panels show the propensity score distributions for the LO-MOGAD and AO-MOGAD groups before and after matching, respectively
(B) For disability risk analysis, the propensity score was estimated with covariates including sex (female), BMI, disease duration, onset phenotype (optic neuritis, myelitis, and brain), EDSS at onset, and acute treatment at onset.
Covariate balance and propensity score distribution for the clinical outcome of moderate disability (N= 350). A total of 101 patients with LO-MOGAD were matched to 101 patients with AO-MOGAD. The left panel shows SMDs of covariates before and after matching, with the dotted line indicating the acceptable threshold (SMD= ±0.2). The middle and right panels depict the propensity score distributions of the LO-MOGAD and AO-MOGAD groups before and after matching, respectively.

© 2026 Ju H et al. *JAMA Network Open*.

eTable 1. Adverse effects of non-steroid immunosuppressant in Patients with Myelin Oligodendrocyte Glycoprotein Antibody-Associated Disease

	LO-MOGAD	AO-MOGAD	MOGAD	P-value
**Azathioprine exposure**	n=57	n=112	n=169	
Any AE, n (%)	14 (24.6)	20 (17.9)	34 (20.1)	.32
Liver enzyme elevation	7	7	14	
Alopecia	3	5	8	
Gastrointestinal symptoms[a]	2	4	6	
Cytopenia	3	1	4	
**Mycophenolate exposure**	n=42	n=80	n=122	
Any AE, n (%)	2 (4.8)	5 (6.3)	7 (5.7)	>.99
Liver enzyme elevation	1	2	3	
Alopecia	0	1	1	
Gastrointestinal symptoms[a]	0	2	2	
Edema	1	0	1	
**IVIG exposure**	n=3	n=19	n=22	
Any AE, n (%)	0 (0.0)	1 (5.3)	1 (4.5)	>.99
Infusion-related reaction	0	1	1	
**Rituximab exposure**	n=6	n=24	n=30	
Any AE, n (%)	0 (0.0)	0 (0.0)	0 (0.0)	NA
**Satralizumab or tocilizumab exposure**	n=1	n=9	n=10	
Any AE, n (%)	0 (0.0)	0 (0.0)	0 (0.0)	NA

Abbreviations: AE, adverse effect; AO-MOGAD, adult-onset myelin oligodendrocyte glycoprotein antibody-associated disease; IVIG, intravenous immunoglobulin; LO-MOGAD, late-onset myelin oligodendrocyte glycoprotein antibody-associated disease; MOGAD, myelin oligodendrocyte glycoprotein antibody-associated disease.

[a]Gastrointestinal symptoms include dyspepsia, nausea, vomiting and diarrhea

© 2026 Ju H et al. *JAMA Network Open*.

eTable 2. Clinical Characteristics of Patients with Myelin Oligodendrocyte Glycoprotein Antibody-Associated Disease According to the Age Groups

	18–39 years (n=161)	40–59 years (n=139)	≥60 years (n=50)	*P* Value	Post-hoc analysis, *P* value		
					18–39 vs 40–59	40–59 vs ≥60	18–39 vs ≥60
**Age at onset, mean (SD), y**	29.7 (6.4)	50.3 (6.2)	66.8 (6.1)	**<.001**	**<.001**	**<.001**	**<.001**
**Sex, n (%)**							
Female	81/161 (50.3)	81/139 (58.3)	27/50 (54.0)	.39			
Male	80/161 (49.7)	58/139 (41.7)	23/50 (46.0)				
**BMI[a], mean (SD), kg/m^2**	23.8 (4.6)	24.9 (3.9)	23.7 (3.0)	.08			
**Disease duration, median (IQR), m**	38.2 (16.0–87.3)	29.8 (11.5–52.0)	21.8 (4.4–32.9)	**<.001**	.09	**.01**	**<.001**
**Attack type at onset, n (%)**							
Optic neuritis	82/161 (50.9)	74/139 (53.2)	27/50 (54.0)	.89			
Myelitis	45/161 (28.0)	34/139 (24.5)	17/50 (34.0)	.42			
Brain[b]	53/161 (32.9)	40/139 (28.8)	8/50 (16.0)	.07			
**Attack type during the course, n (%)**							
Optic neuritis	103/161 (64.0)	83/139 (59.7)	27/50 (54.0)	.42			
Myelitis	62/161 (38.5)	46/139 (33.1)	17/50 (34.0)	.60			
Brain[b]	65/161 (40.4)	50/139 (36.0)	8/50 (16.0)	**.007**	>.99	**.03**	**.006**
**Relapse data**							
Monophasic course, n (%)	56/139 (40.3)	49/112 (43.8)	25/32 (78.1)	**<.001**	.58	**<.001**	**<.001**
Total number of attacks, median (IQR)	2.0 (1.0–3.0)	1.0 (1.0–3.0)	1.0 (1.0–1.0)	**<.001**	.58	**<.001**	**<.001**
ARR[c], median (IQR)	0.14 (0.00–0.38)	0.11 (0.00–0.40)	0.00 (0.00–0.00)	**.008**	>.99	**.02**	**.007**
**Disease disability, EDS score**							
At onset, median (IQR)	3.0 (2.0–4.0)	3.0 (2.0–4.0)	3.0 (2.5–4.5)	.14			
At last follow-up, median (IQR)	1.0 (0.0–2.0)	1.0 (0.0–2.0)	2.0 (1.0–3.125)	**.002**	>.99	**.01**	**.001**
EDSS ≥3 at last follow-up, n (%)	18/161 (11.2)	21/139 (15.1)	14/50 (28.0)	**.02**	.94	0.13	**.01**
EDSS ≥6 at last follow-up, n (%)	2/161 (1.2)	2/139 (1.4)	3/50 (6.0)	.11			
**Visual disability, visual functional system score, median (IQR)**							
At onset, median (IQR)	1.0 (0.0–4.0)	2.0 (0.0–5.0)	3.0 (0.0–5.0)	.17			
At last follow-up, median (IQR)	0.0 (0.0–1.0)	0.0 (0.0–2.0)	1.0 (0.0–2.0)	**.02**	>.99	.08	**.01**
**MOG-IgG**							
Low-positive MOG-IgG, n (%)	14/161 (8.7)	14/139 (10.1)	8/50 (16.0)	.35			

© 2026 Ju H et al. *JAMA Network Open*.

	18–39 years (n=161)	40–59 years (n=139)	≥60 years (n=50)	*P* Value	18–39 vs 40–59		
					18–39 vs 40–59	40–59 vs ≥60	18–39 vs ≥60
Seronegative conversion, n (%)	32/100 (32.0)	20/93 (21.5)	11/28 (39.3)	.11			
**CSF analysis**							
WBC counts, median (IQR), cells/μL	5.0 (1.0–40.0)	4.0 (1.0–15.0)	3.0 (1.0–8.0)	**.03**	.09	>.99	.07
Pleocytosis, n (%)	52/125 (41.6)	33/107 (30.8)	9/39 (17.1)	.06			
Protein, median (IQR), mg/dL	42.8 (30.0–57.9)	40.2 (31.9–58.4)	43.6 (30.8–60.1)	.97			
OCB positive, n (%)	14/119 (11.8)	7/95 (7.4)	0/36 (0.0)	.08			
IgG index, median (IQR)	0.58 (0.49–0.68)	0.55 (0.49–0.64)	0.56 (0.50–0.63)	.65			
**MRI findings**							
**Optic nerve, n (%)**							
Bilateral involvement	31/161 (19.3)	35/139 (25.2)	11/50 (22.0)	.47			
Longitudinal optic nerve involvement	47/161 (29.2)	44/139 (31.7)	20/50 (40.0)	.36			
Perineural optic sheath enhancement	43/161 (26.7)	43/139 (30.9)	18/50 (36.0)	.42			
Optic disc oedema	35/161 (21.7)	40/139 (28.8)	15/50 (30.0)	.29			
**Spinal cord, n (%)**							
Longitudinally extensive myelitis	26/161 (16.1)	23/139 (16.5)	9/50 (18.0)	.95			
Central cord lesion or H-sign	38/161 (23.6)	16/139 (11.5)	7/50 (14.0)	**.02**	.02	>.99	.44
Conus lesion	11/161 (6.8)	5/139 (3.6)	3/50 (6.0)	.46			
**Brain, n (%)**							
Multiple ill-defined T2 hyperintensity lesion in supratentorial and often infratentorial white matter	52/161 (32.3)	29/139 (20.9)	8/50 (16.0)	**.02**	.08	>.99	.08
Deep gray matter involvement	29/161 (18.0)	13/139 (9.4)	6/50 (12.0)	.09			
Ill-defined T2-hyperintensity involving pons, middle cerebellar peduncle, or medulla	46/161 (28.6)	31/139 (22.3)	3/50 (6.0)	**.004**	.65	**.03**	**<.001**
Cortical lesion with or without lesional and overlying meningeal enhancement	24/161 (14.9)	10/139 (7.2)	2/50 (4.0)	**.03**	.11	>.99	.12
**Acute treatment at onset, n (%)**							
Any treatment	132/159 (83.0)	113/138 (81.9)	39/50 (78.0)	.72			
IV steroid	131/159 (82.4)	113/138 (81.9)	39/50 (78.0)	.78			
IV steroid followed by IVIG or PLEX	14/159 (8.8)	7/138 (5.1)	3/50 (6.0)	.43			
IV steroid + IVIG	9/159 (5.7)	3/138 (2.2)	1/50 (2.0)	.23			
IV steroid + PLEX	7/159 (4.4)	4/138 (2.9)	2/50 (4.0)	.79			
IVIG only	1/159 (0.6)	0/138 (0.0)	0/50 (0.0)	>.99			
**Preventive treatment[d], n (%)**							

© 2026 Ju H et al. *JAMA Network Open*.

	18–39 years (n=161)	40–59 years (n=139)	≥60 years (n=50)	*P* Value	18–39 vs 40–59		
					18–39 vs 40–59	40–59 vs ≥60	18–39 vs ≥60
IS use	125/161 (77.6)	103/138 (74.6)	29/50 (58.0)	**.02**	>.99	.08	**.02**
Oral steroids use	27/161 (16.8)	25/139 (18.0)	5/50 (10.0)	.41			
nsIS use	107/161 (66.5)	86/138 (62.3)	24/50 (48.0)	.06			
Azathioprine, mycophenolate	92/161 (57.1)	81/138 (58.7)	24/50 (48.0)	.41			
Rituximab,	17/161 (10.6)	8/139 (5.8)	1/50 (2.0)	.08			
Satralizumab, tocilizumab	6/161(3.7)	1/139 (0.7)	0/50 (0.0)	.10			
IVIG	17/161 (10.6)	4/139 (2.9)	0/50 (0.0)	**.003**	**.03**	>.99	**.04**
**Preventive treatment before relapse[e], n(%)**	61/161 (37.9)	56/139 (40.3)	27/50 (54.0)	.13			
**Treatment-duration-ratio of IS[f], median (IQR)**	0.71 (0.24–0.93)	0.74 (0.20–0.95)	0.57 (0.00–0.90)	.12			
**Treatment-duration-ratio of nsIS[g], median (IQR)**	0.59 (0.15–0.88)	0.46 (0.00–0.90)	0.32 (0.00–0.87)	.23			

Abbreviations: ARR , annualized relapse rate; BMI, body mass index; CSF, cerebrospinal fluid; EDSS, Expanded Disability Status Scale; IgG, immunoglobulin G; IQR, interquartile range; IS, immunosuppressant; IV, intravenous; IVIG, intravenous immunoglobulin; MOG-IgG, myelin oligodendrocyte glycoprotein-immunoglobulin G; MRI, magnetic resonance imaging; nsIS, non-steroid immunosuppressant; OCB, oligoclonal band; PLEX, plasmapheresis; SD, standard deviation; WBC, white blood cell.

[a] BMI data includes 58 missing values.

[b] Brain included acute disseminated encephalomyelitis, cerebral monofocal or polyfocal deficits, brainstem or cerebellar deficits, and cerebral cortical encephalitis.

[c] The evaluation of the ARR was based on patients with a disease duration of ≥12 months. (n=131 in 18–39 years, n=103 in 40–59 years, and n=31 in 87 in ≥60 years)

[d] Preventive treatment was defined as treatment lasting for 3 months or longer.

[e] Preventive treatment before relapse was defined as treatment with immunosuppressant for 3 months or longer, before relapse.

[f] The treatment-duration-ratio of IS was defined as (IS treatment duration)/(overall disease duration).

[g] The treatment-duration-ratio of nsIS was defined as (nsIS treatment duration)/(overall disease duration).

Group comparisons were conducted using the chi-squared or Fisher's exact test for categorical variables and one-way ANOVA or Kruskal–Wallis tests for continuous variables. For pairwise group comparisons, *P*-values were adjusted using the Bonferroni method owing to multiple comparisons.

© 2026 Ju H et al. *JAMA Network Open*.

eTable 3. Baseline Characteristics of Patients with LO-MOGAD and AO-MOGAD with Disease Duration ≥12 Months

	LO-MOGAD (N=87)	AO-MOGAD (N=178)	MOGAD (N=265)	*P* value
**Age at onset, median (IQR), years**	58.2 (54.8–62.2)	33.5 (25.9–40.3)	40.2 (29.7–54.5)	**<.001**
**Female (%)**	52/87 (59.8)	88/178 (49.4)	140/265 (52.8)	.11
**BMI[a], mean (SD), kg/m^2**	24.2 (3.3)	23.9 (4.6)	24.0 (4.2)	.68
**Disease duration, median (IQR), months**	33.7 (24.3–49.7)	50.2 (26.3–93.6)	43.8 (25.1–81.5)	**.001**
**Attack type at onset, n (%)**				
Optic neuritis	47/87 (54.0)	87/178 (48.9)	134/265 (50.6)	.43
Myelitis	24/87 (27.6)	52/178 (29.2)	76/265 (28.7)	.78
Brain[b]	17/87 (19.5)	59/178 (33.1)	76/265 (28.7)	**.02**
**Disease disability, EDSS score**				
At onset, median (IQR)	3.0 (2.0–4.0)	3.0 (2.0–4.0)	3.0 (2.0–4.0)	.26
**Visual disability, visual functional system score, median (IQR)**				
At onset, median (IQR)	2.5 (0.0–5.0)	1.0 (0.0–5.0)	2.0 (0.0–5.0)	.58
**Acute treatment at onset, n (%)**				
Any treatment	71/86 (82.6)	143/176 (81.3)	214/262 (81.7)	.80
**Preventive treatment before relapse[c]**	46/87 (52.9)	63/178 (35.4)	109/265 (41.1)	**.007**

Abbreviations: AO-MOGAD, adult onset myelin oligodendrocyte glycoprotein antibody-associated disease; BMI, body mass index; EDSS, Expanded Disability Status Scale; IQR, interquartile range; LO-MOGAD, late-onset myelin oligodendrocyte glycoprotein antibody-associated disease; MOGAD, myelin oligodendrocyte glycoprotein antibody-associated disease; SD, standard deviation.

[a] BMI data includes 55 missing values.

[b] Brain included acute disseminated encephalomyelitis, cerebral monofocal or polyfocal deficits, brainstem or cerebellar deficits, and cerebral cortical encephalitis.

[c] Preventive treatment before relapse was defined as treatment with immunosuppressant for 3 months or longer, before relapse.

Analyses were performed using the chi-squared or Fisher's exact test for categorical variables, as appropriate. The independent *t*-test or Mann–Whitney U test was used for continuous variables, depending on the normality of data distribution.

© 2026 Ju H et al. *JAMA Network Open*.

eTable 4. Cox Proportional Hazard Regression Analysis for Factors Associated with Relapses in Patients with MOGAD using myelitis onset as a covariate

Variable	Model 1 Adjusted HR (95% CI)	*P* value	Model 2 Adjusted HR (95% CI)	*P* value
**Late onset**	0.64 (0.43–0.95)	**.03**	0.75 (0.50–1.12)	.16
**Onset phenotype**				
Myelitis	0.53 (0.35–0.80)	**.003**	0.52 (0.34–0.79)	**.002**
**Preventive treatment before relapse[a,b]**			0.18 (0.10-0.32)	**<.001**

Abbreviations: CI, confidence interval; HR, hazard ratio; MOGAD, myelin oligodendrocyte glycoprotein antibody-associated disease.

[a] Preventive treatment before relapse was defined as treatment with immunosuppressant for 3 months or longer, before relapse and was treated as a time-dependent covariate.

[b] Preventive treatment before relapse had one missing value.

Adjusted HR for multivariable analysis are presented. Model 1: adjusted for myelitis at onset; Model 2: additionally adjusted for preventive treatment before relapse.
Unadjusted estimates are identical to those in Table 2 and are omitted for brevity.

© 2026 Ju H et al. *JAMA Network Open*.

eTable 5. Binary Logistic Regression Analysis for Factors Associated with Moderate Disability at Last Follow-up using myelitis onset as a covariate

Variable	Adjusted OR (95% CI)	*P* value
**Late onset**	2.62 (1.27–5.40)	**.009**
**Disease duration, months**	1.01 (1.00–1.01)	**.01**
**Onset phenotype**		
Myelitis	5.06 (2.53–10.14)	**<.001**
**EDSS ≥3 at onset[a]**	2.63 (1.14–6.05)	**.02**
**Acute treatment at onset[a]**	0.31 (0.13–0.72)	**.007**

Abbreviations: CI, confidence interval; EDSS, Expanded Disability Status Scale; OR, odds ratio.

[a]There were 35 missing values for EDSS ≥3 at onset and 3 missing values for acute treatments at onset

Unadjusted estimates are identical to those in Figure 1 and are omitted for brevity.

© 2026 Ju H et al. *JAMA Network Open*.

eTable 6. Relapse Outcomes in Late onset and Adult-onset Myelin Oligodendrocyte Glycoprotein Antibody-Associated Disease after Propensity Score Matching

	LO-MOGAD (N=64)	AO-MOGAD (N=64)	*P* value
Monophasic course	46/64 (71.9)	40/64 (62.5)	.84
Total number of attacks, median (IQR)	1.0 (1.0–2.0)	1.0 (1.0–2.5)	.12
ARR, median (IQR)	0.0 (0.0–0.3)	0.0 (0.0–0.2)	.81

Abbreviations: AO-MOGAD, adult-onset myelin oligodendrocyte glycoprotein antibody-associated disease; ARR, annualized relapse rate; IQR, interquartile range; LO-MOGAD, late-onset myelin oligodendrocyte glycoprotein antibody-associated disease.
Analysis using generalized estimating equations was performed.

© 2026 Ju H et al. *JAMA Network Open*.
