# Supplementary material for: Disability and Relapse Risk in Late-Onset Myelin Oligodendrocyte Glycoprotein Antibody–Associated Disease
Source: JAMA Netw Open. 2026 Feb 13;9(2):e2559471. doi: 10.1001/jamanetworkopen.2025.59471 (PMC12905660; doi:10.1001/jamanetworkopen.2025.59471)
Supplement: Supplement 2. — Data Sharing Statement [file jamanetwopen-e2559471-s002.pdf]

Data Sharing Statement

Ju. Disability and Relapse Risk in Late-Onset Myelin Oligodendrocyte Glycoprotein Antibody–Associated Disease. *JAMA Netw Open*. Published February 13, 2026.
doi:10.1001/jamanetworkopen.2025.59471

Data
Data available: Yes
Data types: Deidentified participant data
How to access data: Data are available upon reasonable request to the corresponding author
When available: With publication

Supporting Documents
Document types: None

Additional Information
Who can access the data: researchers whose proposed use of the data has been approved
Types of analyses: upon reasonable request to the corresponding author
Mechanisms of data availability: after approval of a proposal
